# The influence of thyroid hormone medication on intra-therapeutic half-life of ^131^I during radioiodine therapy of solitary toxic thyroid nodules

**DOI:** 10.1038/s41598-022-18170-3

**Published:** 2022-08-17

**Authors:** Christian Happel, Wolfgang Tilman Kranert, Benjamin Bockisch, Amir Sabet, Frank Grünwald, Daniel Groener

**Affiliations:** Department of Nuclear Medicine, University Hospital, Goethe University, Theodor Stern Kai 7, 60590 Frankfurt, Germany

**Keywords:** Thyroid diseases, Medical research

## Abstract

Despite a significantly improved dietary iodine supply, solitary toxic thyroid nodules (STN) are still a common clinical problem in former iodine deficient areas. Radioiodine treatment (RIT) is a well-established therapeutic option with few side effects and high success rates. As radioiodine biokinetics are individual for every patient, the necessary activity has to be calculated individually by a pre-therapeutic measurement of the intra-therapeutic effective half-life (EHL) in a radioiodine uptake test (RIUT). A suppressive medication with triiodothyronine (T3) or tetraiodothyronine (T4) is often needed to suppress uptake in normal thyroid tissue. Therefore, the aim of this study was to quantify the possible influence of this medication on intra-therapeutic radioiodine biokinetics. A cohort of 928 patients with STN undergoing RIUT and RIT was analysed. Patients were subdivided into 3 groups. Group T3: medication with T3 (n = 274), group T4: medication with T4 (n = 184) and group NM: no additional medication (n = 470). The T3 and T4 subgroups were further subdivided depending on the dose of thyroid hormone medication. In order to analyse the influence of thyroid hormone medication on individual intra-thyroidal biokinetics, the variance of the determined individual EHL between RIUT and RIT within the single groups and within the subgroups was investigated. EHL was significantly decreased between RIUT and RIT in the T3 and T4 subgroups (EHL: T3: 5.9 ± 1.1 d in RIUT and 3.3 ± 1.4 d in RIT (− 43%) (*p* < 0.05); T4: 5.9 ± 1.2 d in RIUT and 3.4 ± 1.5 d in RIT (− 42%) (*p* < 0.05). The decrease of EHL did not differ statistically between T3 or T4. However, both showed a highly significant difference compared to the NM group (*p* < < 0.05). A further subgroup analysis showed a significant dependence of the decrease in EHL related to the dose of thyroid hormone medication of 35–58% (T3) and 15–67% (T4) (*p* < 0.05). A significantly reduced EHL compared to RIUT in patients receiving thyroid hormone medication was detected. Moreover, a significant correlation between the dose of thyroid hormone medication (T3 or T4) and the decrease of EHL was found. Therefore, an adaption of the calculated activity should be considered in RIUT to obtain the required radiation dose in RIT of patients suffering from STN.

## Introduction

Dietary iodine supply has significantly improved in most countries of the world during the last decades due to corresponding health policy regulations^1^. However, solitary toxic thyroid nodules (STN) are still a common clinical problem^1,2^. STN occur if regions of the thyroid produce excessive amounts of thyroid hormone independent of the thyroidal regulatory circuit^2–4^. One major factor in the development of STN is long lasting alimentary deficit of iodine, leading to a proliferation of naturally existing autonomous thyroid cells^3–7^. The gold standard in the treatment of STN is radioiodine-131 treatment (RIT) which has fewer side effects and is much more convenient for the patients compared to alternative invasive therapeutic options such as thyroid surgery or local thermal ablative procedures^8–13^.

The underlying mechanism of RIT is the selective accumulation of administrated sodium iodine 131 in the thyroid by sodium iodine symporters (NIS)^3,6,14^. ^131^I decays into stable ^131^Xe by emitting beta particles with a mean energy of 190 keV and a physical half-life of 8 days. However, the effective half-life of the radionuclide in the patient is individually lower, due to excretion by voiding, ventilation and transpiration. Therefore, a radioiodine-131 uptake testing (RIUT) prior to RIT is mandatory to estimate individual intra-thyroidal effective half-life and uptake for each patient^10,11,15^. In several countries, the pre-therapeutic determination of the effective half-life is a non-optional requirement. It directly influences the absorbed radiation dose to the thyroid and therefore the success of RIT^10,11,15,16^. The emitted beta radiation leads to ionisation resulting in cell death.

Autonomic function can be caused e.g. by a TSH-receptor mutation, leading to a permanent activation of NIS. As a consequence, an intracellular dissociation of adsorbed stimulating G-protein occurs, generating cAMP and therefore an increased thyroid hormone production^2–5^. Intratherapeutic protection of the healthy non-autonomous thyroid tissue surrounding the STN requires minimizing its iodine uptake through TSH suppression. The STN may have been initially detected clinically or scintigraphically and subsequently present with low level, but not completely suppressed TSH-values. As set out by Dietlein et al. in the German guideline for radioiodine therapy, temporary exogenous TSH-suppression is warranted if TSH values exceed 0.3 mU/L prior to radioiodine testing^10^. Therefore, the patient receives the synthetically produced thyroid hormones triiodothyronine (T3) or tetraiodothyronine (T4) to reduce the TSH level, leading to a reduction of NIS activity, that is accompanied by a decreased iodine-131 uptake in healthy thyroid tissue^10,11,15,17,18^. In some of the patients, healthy thyroid tissue function is supressed by high endogenous secretion of thyroid hormones by the STN (non-compensated STN). The metabolism of STN is largely independent of the TSH level, thus resulting in a selective irradiation of the autonomous nodule^2,6,19^. However, thyroid hormone medication may influence the biokinetics of radioiodine-131 and therefore the success of RIT^16,20^.

The aim of this study was therefore to evaluate the influence of suppressive thyroid hormone medication on intratherapeutic effective half-life during RIUT and RIT in a retrospective monocentric analysis of patients with STN.

## Material and methods

In a retrospective monocentric study, a total of 1550 patients with STN who underwent both RIUT and RIT between 1999 and 2019 were screened. The study was approved by the local ethic committee. Inclusion criteria were either a constant dose of T3 or T4 medication (compensated STN) or no thyroid hormone medication (non-compensated STN) over a period of at least 1 week prior to RIUT and the complete duration of RIT. Further inclusion criteria was a calculated effective half-life between 2 and 8 days in RIUT as well as in RIT that was obviously not influenced by deficient measurements of the remaining activity in the thyroid. Furthermore, only patients who received a full RIUT over 96 h with measurement of the remaining activity 48 h and 96 h after administration of the test activity were included.

Finally, a cohort of 928 patients (641 females, 287 males; mean age: 61 ± 13 years was examined. These patients were subdivided into three groups. Group T3 consists of 274 Patients (200 f., 74 m; mean age: 59 ± 13 years) with a constant dose of T3 thyroid hormone medication. In group T4, 184 patients (141 f., 43 m; mean age: 59 ± 13 years) with a constant dose of T4 thyroid hormone medication were included. The NM group (no medication) consists of 470 patients (300 f., 170 m; mean age: 63 ± 13 years) who were supressed endogenously and therefore received no additional medication over a period of at least 1 week prior to RIUT and the complete duration of RIT. The T3 and T4 group were further subdivided depending on the dose of thyroid hormone medication. The subgroups for group T3 were T3_20: patients receiving T3 20 (n = 76), T3_40: T3 40 (n = 147); T3_60: T3 60 (n = 46) and T3_80: T3 80 (n = 5) µg/d thyroid hormone medication respectively. Consequently, the subgroups for group T4 were T4_25: patients receiving T4 25 (n = 4), T4_50: T4 50 (n = 44), T4_75: T4 75 (n = 58), T4_100: T4 100 (n = 49), T4_125: T4 125 (n = 11), T4_150: T4 150 (n = 17) and T4_200: T4 200 (n = 1) µg/d thyroid hormone medication, respectively. The success of exogenous suppression was verified by TSH measurement on the first day of RIUT. Table 1 shows the demographic data of the final study cohort (Table 1).

**Table 1:** Comparison of the investigated subgroups regarding demographic and biokinetics data.

Group/subgroup	n	Female	Male	Mean age [years]	Mean thyroid volume [ml]	Mean nodule volume [ml]	TSH in RIUT [mU/l] (n)	Mean EHL RIUT [days]	Mean EHL RIT [days]	Ratio EHLRIT/RIUT	Mean time of hospitalization [days]	Mean administered activity in RIT [MBq]	Number of meassurements during RIT	Mean administered target dose [Gy]
Complete cohort	928	641	287	60.7 ± 13	26.1 ± 14.3	7.83 ± 6.9	0.15 ± 0.42 (582)	5.66 ± 1.2	3.81 ± 1.6	0.69 ± 0.29	2.5	668 ± 289	5.0 ± 1.6	329 ± 160
T3 all patients	274	200	74	58.7 ± 13	23.0 ± 11.4	5.39 ± 4.7	0.20 ± 0.39 (185)	5.86 ± 1.1	3.29 ± 1.4	0.57 ± 0.15	2.5	643 ± 292	5.0 ± 1.2	291 ± 144
T3_20	76	53	23	58.6 ± 14	24.1 ± 11.1	6.39 ± 4.2	0.27 ± 0.46 (49)	5.55 ± 1.1	3.58 ± 1.3	0.65 ± 0.21	2.4	637 ± 283	4.9 ± 1.1	305 ± 126
T3_40	147	106	41	60.1 ± 12	24.2 ± 11.9	5.46 ± 5.3	0.13 ± 0.26 (100)	5.93 ± 1.0	3.46 ± 1.4	0.59 ± 0.23	2.5	653 ± 302	5.0 ± 1.3	302 ± 155
T3_60	46	37	9	55.4 ± 13	17.7 ± 8.1	3.55 ± 2.8	0.34 ± 0.55 (32)	6.17 ± 1.1	2.41 ± 1.3	0.40 ± 0.20	2.6	617 ± 276	5.1 ± 1.0	238 ± 120
T3_80	5	4	1	51.4 ± 5	19.6 ± 9.7	3.42 ± 1.4	0.03 ± 0.02 (4)	5.69 ± 0.2	2.36 ± 1.0	0.42 ± 0.18	2.6	667 ± 250	5.2 ± 0.8	231 ± 98
T4 all patients	184	141	43	58.7 ± 13	21.7 ± 10.3	4.75 ± 3.5	0.10 ± 0.14 (99)	5.90 ± 1.2	3.38 ± 1.5	0.58 ± 0.24	2.2	606 ± 268	4.4 ± 1.2	306 ± 146
T4_25	4	2	2	64.0 ± 5	24.2 ± 11.4	8.65 ± 7.2	0.04 ± 0.03 (2)	6.34 ± 1.2	5.27 ± 1.3	0.85 ± 0.24	3.0	571 ± 349	6.0 ± 0.7	448 ± 249
T4_50	44	32	12	61.4 ± 14	24.5 ± 11.1	5.28 ± 3.2	0.12 ± 0.19 (26)	6.01 ± 1.0	3.82 ± 1.5	0.64 ± 0.21	2.2	588 ± 250	4.3 ± 1.2	322 ± 117
T4_75	58	46	12	61.7 ± 10	20.6 ± 9.2	4.72 ± 3.1	0.10 ± 0.11 (26)	5.92 ± 1.2	3.48 ± 1.6	0.60 ± 0.27	2.1	629 ± 270	4.2 ± 1.3	307 ± 160
T4_100	49	39	10	55.3 ± 14	20.5 ± 11.3	4.44 ± 3.8	0.10 ± 0.14 (26)	5.75 ± 1.3	3.15 ± 1.1	0.57 ± 0.23	2.3	621 ± 287	4.6 ± 1.2	306 ± 140
T4_125	11	8	3	55.0 ± 12	21.5 ± 8.0	3.05 ± 2.2	0.09 ± 0.06 (8)	6.05 ± 1.1	2.82 ± 0.9	0.47 ± 0.15	2.4	527 ± 250	4.8 ± 0.8	278 ± 76
T4_150	17	14	3	53.1 ± 8	20.9 ± 7.6	4.48 ± 3.3	0.06 ± 0.05 (11)	5.71 ± 1.2	2.57 ± 1.0	0.46 ± 0.19	2.3	562 ± 198	4.5 ± 1.1	260 ± 147
T4_200	1	0	1	41.9	25.0	6.3	–(0)	7.24	2.37	0.33	2	1.072	4	115
NM (no medication)	470	300	170	62.6 ± 13	29.6 ± 16.2	10.5 ± 7.53	0.14 ± 0.49 (298)	5.44 ± 1.2	4.28 ± 1.5	0.80 ± 0.30	2.7	707 ± 290	5.3 ± 1.9	359 ± 168

RIUT and RIT were performed in accordance with the German guidelines for RIUT and RIT in their current versions^10,11^. RIUT was performed by administration of a radioiodine-131 capsule (2–4 MBq) 1 week prior to inpatient RIT in order to determine individual extrapolated-maximum-^131^I-uptake (EMU) and effective half-life (EHL). Measurement of the remaining radioiodine-131 activity was performed using an individually calibrated gamma scintillation probe with a connected multichannel analyser 48 and 96 h after administration of the radioiodine-131 test capsule. EHL was calculated using an exponential fit of the time activity curve. Thyroid and nodule volumes were determined prior to TSH suppression via ultrasound by experienced nuclear medicine physicians. The designated target dose to the STN was 400 Gy^13^. The required activity was calculated using an MS Excel template based on the Marinelli equation developed in-house^6,10,15,21^. After oral administration of the RIT capsule, patients were hospitalized for at least 2 days (mean time of hospitalization 2.5 days) (Table 1). During the in-patient stay, measurements of the remaining intra-thyroidal activity were performed twice a day using a calibrated gamma scintillation probe with connected multichannel analyser (mean number of measurements: 5) (Table 1)^6,16^. EHL was again calculated using a mono exponential fit of the time activity curve (Marinelli equation). Patients were discharged after falling below the German regulatory limit of 250 MBq for residual activity in the body^10,11^.

To analyse the influence of thyroid hormone medication on individual intrathyroidal biokinetics the variance of the determined individual EHL between RIUT and RIT within the single groups and within the subgroups as well as the variance of the individual EHL in comparison to the subgroups was investigated. Statistic evaluation was performed using the analysis of variance (ANOVA) for comparison of the different groups. Linear regression was performed to evaluate significance of the dose dependence of changes in the EHL. Statistical significance was indicated with *p*-values < 0.05. Data were collected for descriptive analysis in Microsoft Excel, statistical calculations were performed using GraphPad Prism (Version 9.3.1).

### Ethics approval

This study was performed in line with the principles of the Declaration of Helsinki. Approval was granted by the Ethics Committee of University Frankfurt (18.06.2020/20-777).

### Consent to participate

Informed consent was obtained from all individual participants included in the study.

## Results

The different groups and subgroups were statistically compared regarding age, sex, TSH prior to RIUT and EHL in RIUT. No significant differences could be detected. The analysis of the groups T3 and T4 showed a statistically significant decrease in intra-therapeutic EHL compared to the EHL in RIUT (T3: 5.86 ± 1.09 d in RIUT and 3.29 ± 1.41 d in RIT (− 43%) (*p* <  < 0.05); T4: 5.90 ± 1.18 d in RIUT and 3.38 ± 1.46 d in RIT (− 42%) (*p* <  < 0.05)). For the NM group (patients without any thyroid hormone medication) the mean effective half-life was 5.44 ± 1.2 days in RIUT and 4.28 ± 1.53 days in RIT. This reduction of 21% was statistically significant as well (*p* < 0.05). The determined data for the complete cohort and the subgroups are summarized in Table 1.

**Figure 1:**
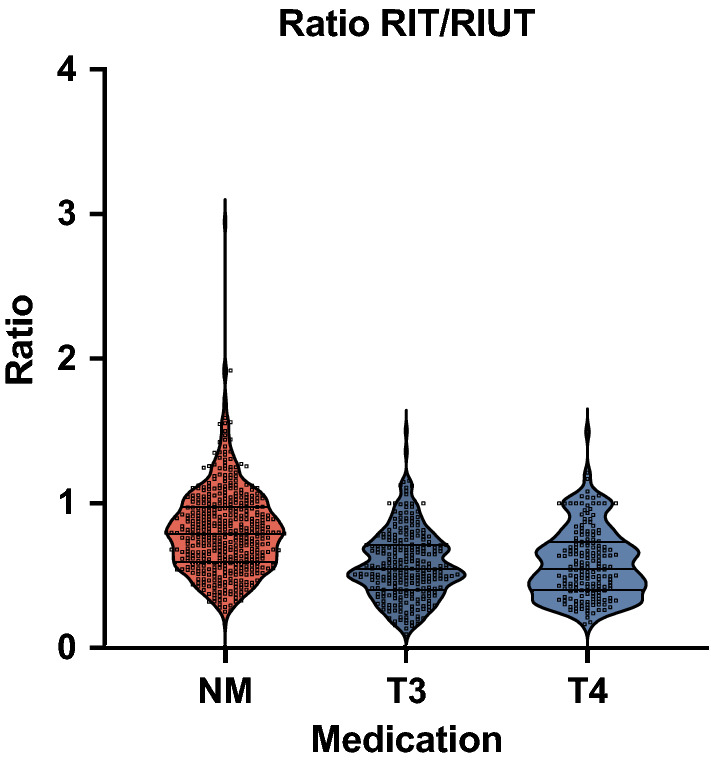
Comparison of the ratio EHL RIT/EHL RIUT in the three investigated groups.

The comparison of the reduction in intra-therapeutic effective half-life between the different groups is shown in Fig. 1. The decrease of EHL for group T3 and group T4 did not differ statistically (*p* = 0.86). However, both of them showed a highly significant difference compared to group NM (patients without any thyroid hormone medication) (*p* < 0.0001). Therefore, the decrease of intra-therapeutic EHL is significantly more pronounced when thyroid hormone medication is administered.

**Figure 2:**
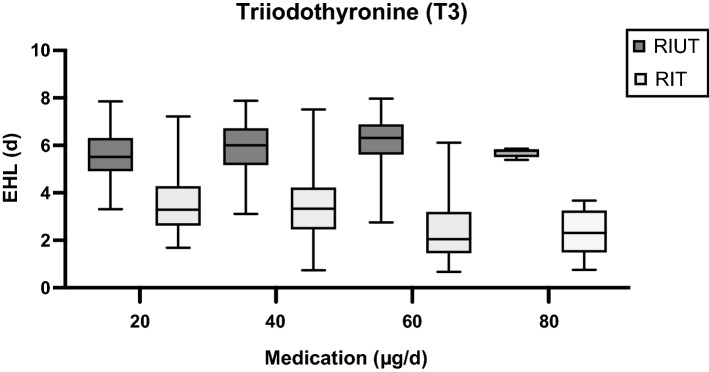
Development of pre- and intratherapeutic EHL depending on the dose of T3.

The comparison of the subgroups T3_20 to T3_60 showed, that there is a highly significant correlation of the decrease of EHL between RIUT and RIT with the dose of T3 thyroid hormone medication (*p* < 0.05). Mean reduction of EHL was 35% for T3 thyroid hormone medication with T3 20 µg/d (T3_20), 41% for T3 40 µg/d (T3_40) and 60% for T3 60 µg/d (T3_60) (Table 1, Fig. 2). In subgroup T3_80 (T3 80 µg/d) the mean decrease of the EHL was 58%. This seems to plateau and may be caused by the low number of patients in this subgroup of only 5. Mean EHL in RIUT did not differ significantly across the subgroups T3_20 to T3_80 (*p* > 0.05). Table 1 and Fig. 2 shows the mean ratio of EHL in RIT and EHL in RIUT and therefore the relative reduction of the EHL between RIUT and RIT.

**Figure 3:**
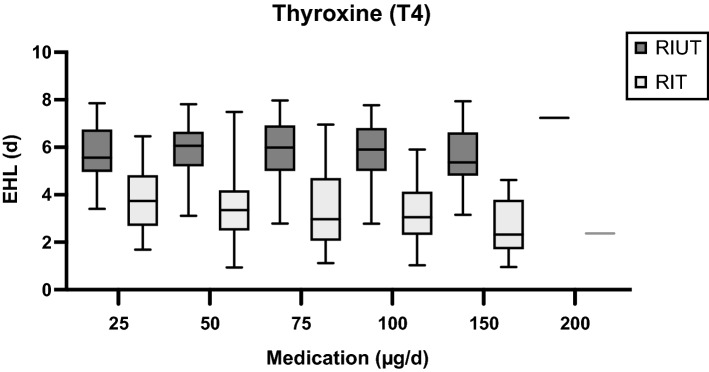
Development of pre- and intratherapeutic EHL depending on the dose of T4.

The comparison of the subgroups T4_25 to T4_200 showed a highly significant correlation of the decrease of EHL between RIUT and RIT with the dose of T4 thyroid hormone medication (*p* < 0.05) as well (Table 1, Fig. 3). Mean reduction of EHL was 15% for T4 thyroid hormone medication with T4 25 µg/d (T4_25), 36% for T4 50 µg/d (T4_50), 40% for T4 75 µg/d (T4_75), 43% for T4 100 µg/d (T4_100), 53% for T4 125 µg/d (T4_125), 54% for T4 150 µg/d (T4_150) and 67% for T4 200 µg/d (T4_200) (Table 1, Fig. 3). Mean EHL in RIUT did not differ significantly in any of the investigated subgroups T4_25 to T4_200 (p > 0.05). Table 1 and Fig. 3 show the ratio of EHL in RIT and EHL in RIUT and therefore the relative reduction of the EHL between RIUT and RIT.

## Discussion

RIT has been a well-established therapeutic procedure for decades. However, some aspects of radioiodine-131 biokinetics are currently still not fully understood. The metabolic behaviour of radioiodine-131 is dependent on many internal and external influencing factors, such as a previous administration of radioiodine-131 or additionally administered medication, such as glucocorticoids^6,16^. Though empirical dosage concepts for RIT have been proven feasible, the use of individual dosimetry through a RIUT is advocated to minimize both the risk of post-treatment hypothyroidism and rates of residual autonomy with recurrent hyperthyroidism^22^. As such, treatment activities tailored to the patient’s individual biokinetics are considered a contribution to minimizing radiation exposure and in some countries even a mandatory regulation^23^. Although the influence of thyroid hormone medication during RIT on intra-thyroidal biokinetics of radioiodine-131 is not fully understood^16,24^. In the presented study a significant correlation between the dose of an administered thyroid hormone medication and intra-therapeutic effective half-life of radioiodine-131 could be statistically shown in a large cohort of patients with STN.

Ingested iodine is reduced to iodide in the gastrointestinal tract and is resorbed in the small intestine. Iodine subsequently accumulates specifically in thyroid follicles^17,18^. Free iodide circulating in the blood circuit is excreted by micturition, transpiration and exhalation. The thyroid hormones T3 and T4 are reduced in the liver. During this process iodine is released and secreted into the small intestine through the gall-bladder. In the small intestine the major part of the iodide is re-absorbed, therefore only little iodine is excreted via faeces^2–5,17,18,25^. In hyperthyroid patients with low-iodine nutrition a relatively low intra-thyroidal iodine pool is present. Therefore, ingested iodine is rapidly incorporated in thyroglobulin and rapidly secreted from the thyroid as T3 and T4. The residual time of iodine in the thyroid and the corresponding EHL are therefore short^3,4,17,18^.

Bockisch et al. found significantly reduced EHL in RIT compared to RIUT of approximately 15% and explained this decrease by a release of iodine as a direct consequence of radiation exposure^20,26^. A comparable dependence was shown in a study by Nüchel et al.^27^. Nüchel et al. evaluated 43 patients suffering from STN undergoing RIUT and RIT with radioiodine-131, and found EHL in RIUT significantly reduced compared to EHL in RIT (*p* < 0.05). The mean reduction was 1.4 days with a correlation coefficient of r = 0.34 (*p* < 0.001) between EHL in RIUT and RIT^27^. The authors even concluded to waive RIUT and use a non-individual standardized EHL^27^. However, different results were obtained by Canzi et al.^28^. The authors found in their study with 79 patients suffering from STN, that EHL in RIT was not significantly altered compared to RIUT^28^. However, Canzi et al. performed RIUT by administration of radioiodine-123. Furthermore, the mean time between RIUT and RIT was 20 days^28^, which may have impaired the comparability of their study to our study and the other cited studies^26–28^.

In the presented study, EHL in RIUT was independent from thyroid hormone medication, whereas intra-therapeutic EHL showed a highly significant correlation with thyroid hormone medication. The EHL of patients with T3 or T4 thyroid hormone medication in RIUT was significantly reduced compared to patients without thyroid hormone medication. Nüchel et al. found a significantly reduced intra-therapeutic EHL in patients without any thyroid hormone medication (endogenous TSH suppression) and also in patients with T4 medication compared to RIUT^27^. However, the deviation in both investigated subgroups was equal. In patients without thyroid hormone medication EHL decreased from 5.3 to 4.4 days (by 0.9 days) and in patients with thyroid hormone medication from 6.0 to 5.2 (by 0.8 days).

The definite reason of the observed decrease of intra-therapeutic EHL remains uncertain. A possible explanation is that T3 and T4 themselves lead to a stimulation of the cell metabolism in the follicle-cells. This would lead to increased iodine biokinetics and therefore a reduced EHL. However, EHL in RIUT as well as in RIT should be reduced in patients with thyroid hormone medication compared to those without thyroid hormone medication. Barbaro et al. found that oral administration of T4 leads to an increased iodine-pool after deiodination in the liver. The authors postulated that this increased iodine pool may influence iodine biokinetics in the thyroid cells^25^. Further possible explanations of a reduced EHL in RIT compared to RIUT may be an insufficient or not regularly taken TSH suppression medication (particularly during out-patient RIUT), mistakes in the positioning of the patient in front of the gamma probe, a flawed calibration of the gamma probe and poor compliance regarding the required fasting.

The duration of pre-therapeutic RIUT is also controversially discussed. Bogner and Czempiel et al.^29^ concluded that a RIUT of 5 days is often too short and may lead to intra-individual variability of radioiodine biokinetics. Van Isselt et al. addressed the same topic and indicated changes of the iodine metabolism rate that might lead to physiologic changes in biokinetics^30^. In the presented study RIUT was conducted with measurements of the remaining intra-thyroidal activity 48 and 96 h after administration of the test capsule. A prolonged time of the RIUT including a late measurement (for example 168 h after administration) was not possible for organizational reasons.

Another possible explanation leading to a reduced intra-therapeutic EHL is the radiation dose administered in RIUT. Depending on EHL, intra-thyroidal uptake and the administered activity, this radiation dose may reach values of up to 10 Gy in the STN^6^. This might cause so-called stunning effects of thyroid cells^4,31^. The cells may therefore be damaged or “stunned” by the obtained dose and not able to accumulate more iodine which might lead to a decreased EHL in RIT. However, if this is the cause of the phenomena described here, the same effect should occur in the no medication group which was actually not the case. However, EHL is shorter in STN due to their increased metabolism compared to healthy thyroid tissue. If the patient is not adequately suppressed, EHL in RIUT is an interaction of EHL from healthy tissue and STN. The radiation dose in the healthy thyroid due to the administered activity in RIUT should lead to a destruction of at least some healthy cells. EHL in RIT may now be decreased due to the changed/modified relation of diseased and healthy thyroid cells. However, if this was the reason for the reduced intra-therapeutic EHL, the relation of accumulated activity in the STN and the surrounding healthy thyroid should be different compared to RIUT. This effect could not be systematically detected in the presented cohort.

The clinical relevance of the presented study is the reduction of administered target dose in RIT, which is directly correlated with the decrease of EHL depending on the dosage of thyroid hormone medication. A complete therapeutic success depends on the administered target dose. The target dose to the STN aimed for in this study was 400 Gy^11^. If 400 Gy cannot be reached, the risk of residual posttherapeutic autonomy increases. The analysis of the administrated target dose showed a mean dose of 329 Gy for the complete study cohort, 359 Gy for the non-medicated group, 291 Gy for the T3 group and 306 Gy for the T4 group (Table 1). As a direct consequence of these findings the designated radioiodine-131 activity should be adjusted to better achieve the desired target dose. The required increase in administered activity is directly proportional to the decrease in EHL between RIUT and RIT found in our study. Therefore, the calculation using the Marinelli equation may be adjusted depending on the existence and the dosage of thyroid hormone medication to consider this effect. However, an adaptation of the Marinelli equation is beyond the scope of this study. Therefore, further evaluation has to be carried out in subsequent scientific investigations.

## Conclusion

The presented results show a significantly reduced intra-therapeutic EHL of radioiodine-131 compared to pre-therapeutic RIUT in patients with STN receiving thyroid hormone medication (T3 or T4), compared to patients without thyroid hormone medication. Moreover, a significant correlation between the dose of thyroid hormone medication (T3 or T4) and the decrease (shortening) of intra-therapeutic EHL of radioiodine-131 could be detected. This effect has not been described before. Therefore, an adaption of the calculated activity should already be considered in RIUT to obtain the required radiation dose in RIT for patients with STN.

## Data Availability

The datasets generated during and/or analysed during the current study are available from the corresponding author on reasonable request.
